# Perspectives on future Alzheimer therapies: amyloid-β protofibrils - a new target for immunotherapy with BAN2401 in Alzheimer’s disease

**DOI:** 10.1186/alzrt246

**Published:** 2014-03-24

**Authors:** Lars Lannfelt, Christer Möller, Hans Basun, Gunilla Osswald, Dag Sehlin, Andrew Satlin, Veronika Logovinsky, Pär Gellerfors

**Affiliations:** 1 Department of Public Health/Geriatrics, Uppsala University, Dag Hammarskölds väg 14 B, 751 85 Uppsala, Sweden; 2 BioArctic Neuroscience AB, Warfvinges väg 35, 112 51 Stockholm, Sweden; 3 Eisai, Inc, 100 Tice Boulevard, Woodcliff Lake, NJ 07677, USA

## Abstract

The symptomatic drugs currently on the market for Alzheimer’s disease (AD) have no effect on disease progression, and this creates a large unmet medical need. The type of drug that has developed most rapidly in the last decade is immunotherapy: vaccines and, especially, passive vaccination with monoclonal antibodies. Antibodies are attractive drugs as they can be made highly specific for their target and often with few side effects. Data from recent clinical AD trials indicate that a treatment effect by immunotherapy is possible, providing hope for a new generation of drugs. The first anti-amyloid-beta (anti-Aβ) vaccine developed by Elan, AN1792, was halted in phase 2 because of aseptic meningoencephalitis. However, in a follow-up study, patients with antibody response to the vaccine demonstrated reduced cognitive decline, supporting the hypothesis that Aβ immunotherapy may have clinically relevant effects. Bapineuzumab (Elan/Pfizer Inc./Johnson & Johnson), a monoclonal antibody targeting fibrillar Aβ, was stopped because the desired clinical effect was not seen. Solanezumab (Eli Lilly and Company) was developed to target soluble, monomeric Aβ. In two phase 3 studies, Solanezumab did not meet primary endpoints. When data from the two studies were pooled, a positive pattern emerged, revealing a significant slowing of cognitive decline in the subgroup of mild AD. The Arctic mutation has been shown to specifically increase the formation of soluble Aβ protofibrils, an Aβ species shown to be toxic to neurons and likely to be present in all cases of AD. A monoclonal antibody, mAb158, was developed to target Aβ protofibrils with high selectivity. It has at least a 1,000-fold higher selectivity for protofibrils as compared with monomers of Aβ, thus targeting the toxic species of the peptide. A humanized version of mAb158, BAN2401, has now entered a clinical phase 2b trial in a collaboration between BioArctic Neuroscience and Eisai without the safety concerns seen in previous phase 1 and 2a trials. Experiences from the field indicate the importance of initiating treatment early in the course of the disease and of enriching the trial population by improving the diagnostic accuracy. BAN2401 is a promising candidate for Aβ immunotherapy in early AD. Other encouraging efforts in immunotherapy as well as in the small-molecule field offer hope for new innovative therapies for AD in the future.

## Introduction

Immunotherapy has emerged as a promising treatment option for Alzheimer’s disease (AD), the most common form of dementia [1]. The lack of an effective treatment is an increasing socioeconomic threat. Although many challenges remain, data from drug programs within the immunotherapy area indicate that treatment effects are possible, providing hope for a new generation of therapies in the future.

The underlying pathogenic mechanism for the development of AD is subject to ongoing discussions. According to the amyloid hypothesis, the amyloid-beta (Aβ) peptide, which is the main constituent of extracellular plaques found in AD brains [2], initiates the disease process and therefore is an attractive target for intervention [3,4]. This hypothesis has been supported by the findings of several mutations in the Aβ region of the amyloid-beta precursor protein (AβPP) as well as in other genes in families with autosomal dominant, early-onset AD [5-9]. The mutations have been shown to increase the production of Aβ *in vitro* as well as *in vivo* (reviewed in [3,10]). The Arctic mutation (*AβPP E693G*) points to large soluble Aβ oligomers (that is, protofibrils) to be toxic and driving the disease process. We found that the Arctic Aβ peptide had a propensity to form large soluble Aβ protofibrils [8], and later studies on AD cases with the Arctic mutation indeed showed that they are negative for fibrillized amyloid, as measured by binding of Pittsburg compound B (^11^C-PIB) to brain amyloid with positron emission tomography (PET) [11]. However, in the most prevalent form of the disease, late-onset sporadic AD, decreased Aβ clearance rather than increased production is initiating the disease process [12]. A recent finding of a protective mutation in the *AβPP* gene (A673T) resulted in reduced β-secretase cleavage of AβPP as well as a lowered risk of developing sporadic AD and slowing of the rate of cognitive decline in an older population [13], further strengthening the amyloid hypothesis.

The lack of effect on disease progression in AD by the symptomatic drugs currently on the market creates a large unmet medical need. Many new candidate drugs are targeting the production, aggregation, or clearance of Aβ such as γ-secretase inhibitors [14,15] and β-secretase inhibitors [16-18]. Other interesting approaches are small molecules targeting pyroglutamated toxic Aβ peptides [19] or aggregated Aβ [20,21].

## Amyloid-beta immunotherapy

Biopharmaceuticals constitute the class of drugs that has developed most rapidly during the last decade. These drugs include monoclonal antibodies and molecules stimulating the patient’s own immune system. A number of immunotherapy programs for AD aimed at lowering the amount of Aβ in brain have evolved. Immunotherapy targeting Aβ has emerged as an attractive approach for disease intervention in AD, as Aβ immunotherapy in general confers a lower risk of side effects in a vulnerable patient population during long-term treatment as compared with small-molecule anti-Aβ therapy. However, Aβ immunotherapy is not without side effects, as has been seen in the AN1792 trial [22] with meningoencephalitis in some patients and vasogenic edema or microhemorrhages (or both) in the Bapineuzumab trial [23]. One advantage with antibodies is that they can be made with high specificity for its target, and antibodies usually have a more favorable safety profile than small molecules. Importantly, results from some late-phase anti-Aβ immunotherapy studies indicate that positive effects in the clinic are possible, which is encouraging for continued research.

The two approaches most used in immunotherapy are active and passive immunization. Active immunization includes administration of an antigen to increase the immune response and generate antibodies in the recipient. The advantage of this approach is that it could give a long-term response, requiring fewer administrations of the drug, and also the cost of goods is low. A disadvantage could be that the polyclonal response has a varying amount and specificity of the antibodies generated, in some cases not generating meaningful titers. Especially in an older population such as the late-onset AD group, age-related attenuation of the immune system will affect the efficacy of active immunotherapy. The specificity of the generated antibodies can be difficult to predict, and adverse reactions may be persistent and difficult to treat. In passive immunization, externally generated antibodies are injected into the recipient. These antibodies can be donor-derived human polyclonal antibodies or can be humanized monoclonal antibodies. The advantage of the latter approach is that it allows precise targeting of epitopes. The disadvantage is that it requires frequent intravenous (i.v.) or subcutaneous administrations. For the future, long-term prevention of AD seems more feasible with an active vaccine; however, this requires very adequate biomarkers to know how to select the patients.

## Previous data from clinical amyloid-beta immunotherapy programs

The development of the vaccine AN1792 by Elan (Dublin, Ireland) started when it was observed that immunization of AβPP transgenic mice with fibrillar Aβ in combination with an adjuvant led to formation of anti-Aβ antibodies and clearance of existing amyloid deposits and also prevention of the formation of new deposits. AN1792 was halted in phase 2 because of aseptic meningoencephalitis in 6% of the treated patients [22]. The clinical outcomes were not improved in the active group as compared with the placebo group. However, postmortem examinations of brains from several study participants receiving the drug demonstrated fewer amyloid deposits than would be expected in patients at such a late stage of the disease, indicating that AN1792 had reached its target [24]. In a follow-up study performed 4.6 years after the immunizations were conducted in the phase 2 study, previously identified antibody responders were compared with placebo-treated patients [25]. The antibody responders maintained a low antibody titer and demonstrated significantly reduced cognitive decline compared with placebo-treated patients, supporting the hypothesis that Aβ immunotherapy may have long-term effects.

Passive i.v. immunization in the program for Bapineuzumab (Elan/Pfizer Inc., New York, NY, USA/Johnson & Johnson, New Brunswick, NJ, USA), a monoclonal antibody targeting fibrillar Aβ and directed against Aβ1-5, was stopped in 2012 after failing to reach the clinical endpoint in phase 3. Interestingly, Bapineuzumab treatment lead to a small but significant reduction of total tau as well as phospho-tau in cerebrospinal fluid (CSF) [26], indicating a reduction of neural loss. The levels of Aβ in CSF did not differ between Bapineuzumab- or placebo-treated patients. In a separate study in 28 patients with AD, the amyloid load was found to be reduced in the brains of patients treated with Bapineuzumab as compared with placebo, as measured by binding of ^11^C-PIB to brain amyloid with PET [27]. Bapineuzumab treatment was associated with vasogenic edema called amyloid-related imaging abnormalities with parenchymal edema as well as intracerebral microhemorrhages. This could be the result of antibodies binding and dissolving aggregated Aβ in brain tissue as well as in blood vessel walls, where a local reaction may lead to impairment of the blood–brain barrier. The adverse event profile resulted in a lowering of the dose, and the desired clinical effect was not achieved. This led to the termination of the i.v. program. One possible explanation for these observations is that the drug was given too late in the disease progression or that, owing to misdiagnosis, the trial population was not sufficiently enriched. Alternatively, the dose was too low because of safety concerns.

Solanezumab (Eli Lilly and Company, Indianapolis, IN, USA) was developed to target the mid-region of soluble, monomeric Aβ. In a phase 2 study of Solanezumab in mild to moderate AD, a dose-dependent increase in CSF Aβ_42_ was observed. No effect was found on CSF tau, amyloid PET, hippocampal volume, or Alzheimer Disease Assessment Scale-cognitive subscale (ADAS-Cog) [28]. In two phase 3 studies, Solanezumab failed to meet primary clinical endpoints [29]. However, when data from the two studies later were pooled, a positive pattern emerged, revealing a significant slowing of cognitive decline in the subgroup of mild AD. In addition, a significant improvement was seen in functional scores.

## Ongoing clinical programs: active amyloid-beta immunotherapy

Several active immunotherapy programs have reached clinical phase, as listed in Table 1. Affitope AD02 is designed to induce antibody production without T-cell activation, as T cells were seen in patients with meningoencephalitis in the AN1792 trial. It has been reported to meet primary safety and tolerability endpoints in phase 1. CAD106 targets Aβ oligomers and has met the primary safety and tolerability endpoints in a third phase 2 study, after multiple subcutaneous injections in patients with mild AD [30]. Additional active immunotherapy programs in early clinical development are listed in Table 1.

**Table 1:** Ongoing and terminated active amyloid-beta immunotherapy clinical programs in Alzheimer’s disease

**Name**	**Company**	**Phase**	**Antigen**
Affitope AD02	Affiris/GlaxoSmithKline	2	Aβ_1–6_
CAD106	Novartis	2	Aβ_1–6_
Vanutide cridificar	Elan/Johnson & Johnson/Pfizer Inc.	2	Aβ_1–6_
ACI24	AC Immune/Bayer Healthcare Pharmaceuticals	1/2	Aβ_1–15_
V950	Merck & Co.	1	Not published
Affitope AD03	Affiris/GlaxoSmithKline	1	Pyroglutamate modified Aβ
UB311	United Biomedical	1	Aβ_1–14_
AN1792	Elan	Terminated	Aβ_1–42_

Aβ, amyloid-beta.

## Ongoing clinical programs: passive amyloid-beta immunotherapy

Likely owing to the challenges with active immunization described above, passive Aβ immunization programs are currently more numerous (Table 2). Eli Lilly and Company has announced that a new phase 3 study in patients with mild AD will be performed with Solanezumab, and the antibody has also been selected for evaluation in prodromal familial AD in the Dominantly Inherited Alzheimer Network (DIAN) Trial and the Anti-Amyloid Treatment in Alzheimer’s Disease Prevention Trial (A4), as described below. Gantenerumab (Roche, Basel, Switzerland), also in the DIAN trial, is intended for use in prodromal AD and is currently in phase 2/3 of clinical development, and it targets a combination of the N-terminal and mid-regions of Aβ. BAN2401 (Eisai, Tokyo, Japan/BioArctic Neuroscience, Stockholm, Sweden) selectively targets soluble Aβ protofibrils and is currently in phase 2b, having shown a favorable safety profile in earlier studies. Crenezumab (Genentech, South San Francisco, CA, USA/Roche) targets oligomeric and fibrillar forms of Aβ in mild to moderate AD and is in phase 2 of clinical development as well as in the Alzheimer’s Prevention Initiative (API), as described below. Additional passive anti-Aβ immunotherapy programs in early clinical development are listed in Table 2.

**Table 2:** Ongoing and terminated passive immunotherapy clinical programs in Alzheimer’s disease

**Name**	**Company**	**Phase**	**Trial population**	**Binding domain**	**Target**
Solanezumab	Eli Lilly and Company	3	Prodromal and mild AD	Aβ_16–23_	Soluble Aβ
Gantenerumab	Roche	2/3	Prodromal and mild AD	Combined Aβ N-terminal and mid domain, conformational	Aggregated Aβ
BAN2401	Eisai/ BioArctic Neuroscience/Eisai	2b	MCI due to AD or mild AD	N-terminal, conformational	Soluble Aβ protofibrils
Crenezumab	Genentech/Roche	2	Prodromal and mild/moderate AD	Aβ 12-23	Soluble oligomeric/fibrillar Aβ and plaque
Bapineuzumab	Elan/ Pfizer Inc./Johnson & Johnson	Intravenous and subcutaneous programs terminated	Mild/moderate AD	Aβ_1–5_	Soluble and aggregated Aβ
BIIB037	Biogen Idec/Neuroimmune Therapeutics	1	MCI due to AD or mild AD	Conformational Aβ	Fibrillar Aβ
AAB003	Elan/Pfizer Inc./Janssen	1	Mild/moderate AD	Aβ_1–6_	Soluble and aggregated Aβ
SAR228810	Sanofi	1	Mild/moderate AD	Not published	Soluble oligomeric/protofibrillar Aβ
ABP102	Abiogen Pharma	1	AD	Catalytic antibody cleaving Aβ	Aggregated Aβ
Ponezumab^a^	Pfizer Inc.	1	Mild/moderate AD	Aβ_33–40_	Soluble and aggregated Aβ

^a^In phase 2 in congophilic amyloid angiopathy. Aβ, amyloid-beta; AD, Alzheimer’s disease; MCI, mild cognitive impairment.

## Problems with current trials

The recent setbacks with many anti-amyloid small molecules and immunotherapies do not necessarily mean that Aβ is the wrong target for AD treatment. In the recent late-phase failures, a subset of the trial population with mild to moderate AD is likely misdiagnosed, as the clinical AD diagnosis is difficult to make. It is also possible that the severity of disease in the trial population did not allow for clinical improvement (that is, the treatment was given too late in the disease progression) or that instruments for measurement of effect were not sensitive enough. The poor clinical outcome could also be caused by low dosing due to safety findings that limited the dose ranges.

Even though Aβ has remained the focus of AD research since the peptide was found to be the main constituent of senile plaques, it also has been shown that the amyloid plaque density in brain in fact does not correlate with the severity of dementia [31-34]. However, during the 1990s, several research groups showed that neuronal injury was caused by soluble aggregated Aβ species [35,36]. Soluble Aβ is thus an interesting target for AD disease-modifying treatment. However, as soluble Aβ can be anything from monomers to large protofibrils, correct target identification requires a profound understanding of Aβ toxicity.

## Improving amyloid-beta immunotherapy - protofibrils: a new drug target

During the aggregation of monomeric Aβ to insoluble fibrils, an intermediate species that is called protofibrils and that was first described by Walsh and colleagues [37] in 1997 is formed. Using synthetic Aβ peptide, protofibrils have been defined as large (>100 kDa) soluble oligomeric species appearing as a peak in the void volume of a size exclusion chromatography system with a Sephadex G75 column [8,37]. These protofibrils have been shown to induce electrophysiological changes and cause neurotoxicity in rat cortical neurons [38] and inhibit long-term potentiation in mouse hippocampus [39]. Aβ_42_ protofibrils have been shown to induce an inflammatory process through microglial activation *in vitro*, an effect not seen by insoluble fibrils [40].

The sizes and assembly states of the soluble protofibrils have been investigated, and several oligomers of various sizes have been identified in human brain and in brain from *AβPP* transgenic mice [41-44]. One of the AβPP mutations causing early-onset familial AD, the Arctic mutation (*AβPP E693G*), has been shown to specifically increase the rate of formation of protofibrils [8,45,46]. Furthermore, the mutation has been shown to facilitate early intraneuronal Aβ aggregation and protofibril formation, followed by plaque formation in tg-ArcSwe mice [47,48]. Cognitive deficits were shown to occur concomitantly with the formation of intracellular Aβ deposits but before plaque formation in the transgenic mice [48]. The levels of protofibrils in brain, but not the levels of total Aβ, correlated with spatial learning, adding further evidence to the theory that soluble protofibrils are the toxic species [49]. The pool of toxic Aβ species was shown to consist of molecules in the size range of 80 to 500 kDa [44]. The toxic species were detected by mAb158, a protofibril-selective antibody with low binding to monomers and aggregated insoluble Aβ. mAb158 was isolated by using an inhibition enzyme-linked immunosorbent assay in which the antibody and the antigen reacted in solution and in which the selectivity for protofibrils could be detected. In immunohistochemistry, mAb158 detects Aβ in plaques and in the vasculature of AD brains because of the massive amount of Aβ in these structures [43].

In light of the findings described above, Aβ protofibrils are interesting as targets for AD immunotherapy. Transgenic mice carrying both the Swedish and the Arctic mutation were treated with mAb158. mAb158 did not affect the levels of insoluble Aβ in the brains of plaque-bearing mice, whereas it prevented plaque formation if treatment began before the appearance of senile plaques. In both cases, soluble Aβ protofibril levels were diminished [50], showing that mAb158 can selectively reduce protofibrils *in vivo*. A humanized version of mAb158—BAN2401, developed by BioArctic Neuroscience—has binding characteristics essentially indistinguishable from those of mAb158 with at least a 1,000-fold higher selectivity for protofibrils compared with monomers (manuscript in preparation) and 10 to 15 times less binding to fibrils as compared with protofibrils [44]. BAN2401 has now entered a clinical phase 2b trial, as described below.

## Going forward - how can the outcomes of clinical trials be improved?

Many of the anti-Aβ agents tested in humans have been proven to reach their target as shown by measurement of biomarkers. Yet none of them has been able to show convincing and significant clinical improvement. Lessons learned from Bapineuzumab, in which clinical improvement was not seen despite demonstrated target engagement, raise questions regarding target relevance, heterogeneity of the patient population, and timing of drug administration with disease progression. Furthermore, it is possible that the effect markers were not sensitive enough and the exposure was too low because of limitations by the safety profile.

## Defining the optimal trial population

The patients included in clinical trials have traditionally been diagnosed as mild to moderate AD. Targeting Aβ even at this stage of the disease might be too late. Several studies have suggested that the levels of soluble Aβ are increased very early in disease progression and even precede the clinical symptoms [51]. The ideal target population for disease-modifying treatments, such as immunotherapy, could therefore be early AD (that is, mild cognitive impairment (MCI) due to AD and mild AD). Currently, there are no diagnostic biomarkers that are sensitive and specific enough to detect these early patients with sufficient diagnostic accuracy [52]. Only approximately 60% of patients with memory deficits or MCI have actually converted to AD after 10 years, and the annual conversion rate was 5% to 10% [53]. The high number of patients necessary and long study duration needed for performing clinical trials in this population would therefore be unrealistic. Similarly, results from the Bapineuzumab trials suggest that as much as 30% of the patients enrolled in studies did not have an AD diagnosis [54]. The diagnostic accuracy can be improved by scanning enrolled subjects for brain amyloid by PET [55] and excluding subjects who do not fulfill the criteria for amyloid load in the brain. This is being done in the ongoing phase 2b study with BAN2401 (Eisai/BioArctic Neuroscience) and phase 1 study with BIIB037 (Biogen Idec, Weston, MA, USA). Amyloid PET is also a potential marker of disease progression, which is being evaluated in several trials. The measurement of CSF biomarkers such as Aβ_42_ and tau is another aid in the refinement of the clinical diagnosis [52], and they are being explored as markers of disease progression in several trials. By refining the patient population, treatment effects are more likely to be detected and thereby smaller sample sizes can be used. To shed more light on the preclinical events in AD and to obtain further regulatory support for the validity of biomarkers for both diagnosis and disease progression, three prospective longitudinal investigations are now under way: the A4 trial, API trial, and the DIAN trial. The API and DIAN trials are performed in families with autosomal dominant inherited mutations. In addition to validating the preclinical phase of AD and potential biomarkers, potential disease-modifying drug candidates will be included in the programs: Crenezumab in the API program, Gantenerumab in the DIAN program, and Solanezumab in the DIAN and A4 programs.

## Improving the cognitive measurements

The lack of correlation between markers of target engagement and clinical outcome measures is still an unsolved issue in AD trials, which mirrors the knowledge gap of the disease pathogenesis. Disease progression is traditionally monitored by a combination of techniques measuring physical features such as brain atrophy (volumetric magnetic resonance imaging) and neuronal loss or dysfunction (fluorodeoxyglucose PET and functional magnetic resonance imaging). Stable and sensitive instruments to measure subtle cognitive changes in MCI due to AD as well as in early AD are not yet in place. The methods used for cognitive outcome measures which are approved as effect markers by regulatory agencies are often not sensitive enough for patients with early AD. Eisai has recently developed a new, more sensitive cognitive composite scale -Alzheimer Disease Composite Score [56,57], derived from ADAS-cog, Mini-Mental State Exam, and Clinical Dementia Rating-Sum of Boxes - and this is used in the ongoing phase 2b study with BAN2401.

## Improving biomarkers

In terms of biomarkers, there is currently a lack of understanding of the direction and magnitude of change needed to demonstrate a clinical effect [51,58]. Protofibrils/oligomers in CSF are interesting potential AD biomarkers. Currently, CSF tau and Aβ_1–42_ as well as amyloid PET are used predominantly as an aid for the diagnosis of AD and only as exploratory markers of disease progression. Many new biomarkers are currently being investigated, providing hope for new biomarkers and predictors of conversion to dementia in the near future.

## Finding the right dose and exposure

Setting the right dose in clinical immunotherapy trials is difficult. The long half-life of the antibodies, in combination with the lack of sensitive and stable effect markers, makes dosing challenging. In the ongoing BAN2401 trial, Eisai and BioArctic Neuroscience have chosen an adaptive Bayesian design [59]. This way, the key endpoint in the study is continuously monitored in a blinded manner, and the number of patients in the different treatment arms can be adjusted to optimize the size and duration of the study. The design contains six treatment arms in combination with several planned interim analyses, enabling greater allocation of patients to the treatment arms that appear to be showing the greatest efficacy. In this way, the clinical trial design is optimized for finding the right dose regimen faster.

## A beneficial safety profile

Bapineuzumab and several other early Aβ immunotherapy programs have encountered safety issues causing the programs to terminate. Triggering the immune system can cause undesired effects. A beneficial safety profile allowing efficient dose levels without undesired side effects is pivotal for success. Many lessons have been learned from failed or terminated immunotherapy programs, and the safety profiles of Aβ immunotherapy drugs are continuously improving. BAN2401 is in early clinical development, and so far no safety concerns have been raised.

## Conclusions - BAN2401 in clinical development

Aβ immunotherapy has gained a lot of attention and emerges as one of the most attractive approaches for disease intervention in AD. Aβ neurotoxicity has been shown to be caused by soluble protofibrils rather than insoluble fibrils, and this highlights protofibrils as targets for immunotherapy. Preclinical and clinical data on mAb158/BAN2401 suggest that the antibody targets an Aβ species found to be toxic in a clinical setting as well as in preclinical experiments. Results from previous immunotherapy trials have indicated the importance of targeting early AD, and therefore amyloid PET is used in the ongoing BAN2401 phase 2b trial to identify an early patient population. In the same study, a novel sensitive clinical composite score is being used to monitor disease progression and drug effects. An adaptive study design will allow an optimized number of patients and dose arms in the study. When progressing a chronic treatment into a vulnerable patient population, safety and convenience will be key for a successful treatment. BAN2401 is a promising candidate for Aβ immunotherapy in early AD, according to preclinical and clinical data. Other encouraging efforts in immunotherapy as well as in the small-molecule field offer hope for new innovative therapies for AD in the future.

## Note

This article is part of a series on Immunotherapy in Alzheimer’s disease, edited by Philip Scheltens. Other articles in this series can be found at http://alzres.com/series/immunotherapy.

## Abbreviations

11C-PIB: Pittsburg compound B; A4: Anti-amyloid treatment in alzheimer’s disease prevention trial; AD: Alzheimer’s disease; ADAS-Cog: Alzheimer disease assessment scale-cognitive subscale; API: Alzheimer’s prevention initiative; Aβ: amyloid-beta; AβPP: Amyloid-beta precursor protein; CSF: Cerebrospinal fluid; DIAN: Dominantly inherited alzheimer network; i.v.: intravenous; MCI: Mild cognitive impairment; PET: Positron emission tomography.

## Competing interests

LL and PG are founders of BioArctic Neuroscience. GO is chief executive officer, CM is chief security officer, and HB is chief medical officer. AS is head of clinical development for neuroscience and general medicine at Eisai. VL is the project leader for the BAN2401 program at Eisai. DS declares that he has no competing interests.
